# The Alignment of Recommendations of Dietary Guidelines with Sustainability Aspects: Lessons Learned from Italy’s Example and Proposals for Future Development

**DOI:** 10.3390/nu15030542

**Published:** 2023-01-20

**Authors:** Laura Rossi, Marika Ferrari, Andrea Ghiselli

**Affiliations:** 1 Council for Agricultural Research and Economics—Research Centre for Food and Nutrition (CREA–Food and Nutrition), Via Ardeatina 546, 00178 Rome, Italy; 2 Italian Society of Food Science and Nutrition, Via Bu Meliana, 00195 Rome, Italy

**Keywords:** dietary recommendations, food policy, consumers acceptance, sustainability, Italy

## Abstract

The main objective of this paper is to describe the process of the inclusion of sustainability in the Italian Dietary Guidelines (IDGs). In the IDGs’ sustainability chapter, particular emphasis was put on the selection of foods, recommending a plant-based diet with a large quota of vegetable proteins. Advice was also given on the selection of local seasonal products, with low growth input, such as fertilizers, artificial light and heating. Reduction of animal food was recommended, to be substituted with lower impact foods (poultry, milk, eggs and Mediterranean fish including aquaculture). Food waste was largely addressed. Recommendations were made for food purchase planning, food storage and the reuse of leftovers as strategies to reduce waste and save money. The IDGs sustainability recommendations were related to the 16 guiding principles of a sustainable healthy diet and their contribution to the achievement of the Sustainable Developing Goals was provided. The inclusion of sustainability in dietary guidelines requires a multidisciplinary approach to cover the wide range of aspects of a sustainable diet. In the IDGs, it was possible to show that practical recommendations for improving sustainability behavior can be passed on to consumers. Methodological aspects for developing recommendations are not definitive.

## 1. Introduction

Diet composition and food quality have direct effects on human health; however, the indirect health effects caused by environmental change associated with the processes of producing food are less recognized. As national dietary guidelines provide advice for a healthy diet, they must consider both direct and indirect health consequences of nutritional recommendations [1].

In April 2016, the United Nations Decade of Action on Nutrition (2016–2025) was established to recognize the importance of ending all forms of malnutrition, with policies and programs aimed at developing sustainable food systems and promoting healthy dietary practices [2]. In particular, the objectives of the Decade of Action on Nutrition are to support the fulfilment of the Second International Conference on Nutrition (ICN2) commitments [3] and achieve Global Nutrition and diet-related Non-Communicable Disease (NCD) targets by 2025 [4], as well as to contribute to achieving the Sustainable Development Goals (SDGs) by 2030 [5].

Current food systems need to be reshaped to provide health-promoting quality products with a low environmental impact [6]. Coherent actions and innovative food systems are needed to ensure access to sustainable, balanced and healthy diets for all [7]. The quality and sustainability of food systems are also central to fighting against NCDs, considering that isolated interventions have had a limited impact [8]. Efficient approaches include actions for sustainable food systems that promote healthy and safe diets, with national policies integrating nutrition and food safety objectives into food and agriculture policies; strengthening local food production and processing, especially by smallholder and family farmers and developing guidelines on food safety and quality [5]. A healthy diet can be a good entry point for individual and collective action aimed to improve the ability of food systems to provide sufficient affordable and nutritious foods and preserve biodiversity at the production level [9].

Aligning the food-based dietary guidelines (FBDGs) with the latest evidence, not just on healthy eating, but also on the wider social and environmental implications of dietary choices is a key aspect for enabling policy coherence and building a food environment that contributes to public and personal health, as well as promoting local and global environmental sustainability [10,11,12]. The inclusion of sustainability in the FBDGs is in progress in several countries. According to James-Martin et al., 2022 [13], the dietary guidelines of 37 countries included the mention of environmental sustainability, representing approximately 17% of the world’s population. Approximately half (46%), 17 out of these 37 countries, referred to keywords associated with environmental sustainability in their consumer documents and 32 countries (86%) included sustainability aspects in background documents. Countries from Europe and Central Asia were more likely to have referred to environmental sustainability in their FBDG documents than countries in South Asia, East Asia, and the Pacific regions. The FBDGs referred to environmental sustainability mainly in upper-middle-income and high-income countries (34 out of 72), while in low-income or lower-middle-income countries only three (27%) out of the 11 guidelines addressed the issue of sustainability. The inclusion of environmental sustainability messaging was a recent approach since nine out of 10 countries’ FBDGs published since 2019, have included recommendations associated with sustainability that were absent in those published prior to 2010. According to Springmann et al., 2020 [14], the inclusion of sustainability in national dietary guidelines, as well as in World Health Organization’s (WHO) guidelines, could be beneficial, not only from a health perspective, but also necessary for meeting global sustainability goals [5].

The Italian dietary guidelines (IDGs) are explicitly based on the Mediterranean diet, a model that combines consolidated health aspects, acceptance by consumers, and sustainability for production systems, as recently pointed out [15]. The fourth revision of the IDGs, published in 2019, includes a review of evidence and recommendations developed by an independent committee of Italian scientists responsible for preparing the background scientific dossier [16], representing the cultural basis for developing the IDGs [17]; which is a detailed policy document addressed to a large audience. This process of developing the IDGs, the main recommendations, differences to previous revisions, and concordance and differences to international guidance on a healthy diet were described in Rossi et al., 2021 [18]. In brief, a national commission was made responsible for the IDG development. The IDGs included 13 directives divided into four conceptual blocks: (i) how to keep to a balanced weight; (ii) foods to be promoted; (iii) foods to be limited; and (iv) how to ensure a varied and sustainable diet. In the framework of the fourth conceptual block, the editorial coordination board included directive no. 13 for the first time, which focused on the sustainability of food choices.

Hence the main objective of this paper is to describe the process of including sustainability of dietary recommendations in the IDGs. The intended scope of the present work is to show how recommendations for food consumption patterns that are nutritionally sound and support human health were coupled and aligned with food advice that is more sustainable and protective of the environment. The specific purposes of the paper were to compare the IDGs’ sustainability recommendations with the 16 FAO guiding principles of a sustainable healthy diet [3], taking inspiration from the work of James-Martin et al. (2022) [13] and the contribution of the IDGs sustainability chapter to achieve the SDGs, inspired by the work of Grosso et al., 2020 [19].

The policy and research questions underlying the development of this paper were: (i) To what extent would it be possible to develop recommendations combining the capacity of diet to fight obesity and prevent NCDs with sustainable food choices? (ii) What are the actionable policy options that could be put in place to pursue a modification of current food systems, making them sustainable and beneficial for environmental health? (iii) What are the limits to including sustainability in nutritional recommendations and in what way can dietary guidelines contribute to creating a healthy food environment?

## 2. Sustainability Recommendations in the IDGs

### 2.1. The IDGs Sustainability Chapter—Directive 13

As reported by Rossi et al., 2022 [18], one of the novelties of the fourth revision of the IDGs was the inclusion of a chapter with recommendations on the sustainability of food choices. The objective of the sustainability chapter—directive 13 of the Italian guidelines—was the fostering of food choices aimed to promote both human health and environmental protection. The development of the sustainability recommendations was carried out, based on published evidence, statements and data supported by the consensus literature. A multistep approach was adopted with an initial literature study including the use of peer-reviewed papers and technical reports from intergovernmental agencies, non-governmental organisations and private bodies to prepare background documentation. The background scientific dossier provided the emerging issues on sustainability used for the preparation of the sustainability chapter of the guidelines. The second phase of directive 13 development involved discussions about the background document by the coordination committee to define how the main issues could be translated into the guideline addressed to consumers. The third phase was the preparation of the guidelines and their final approval for consensus.

**Figure 1:**
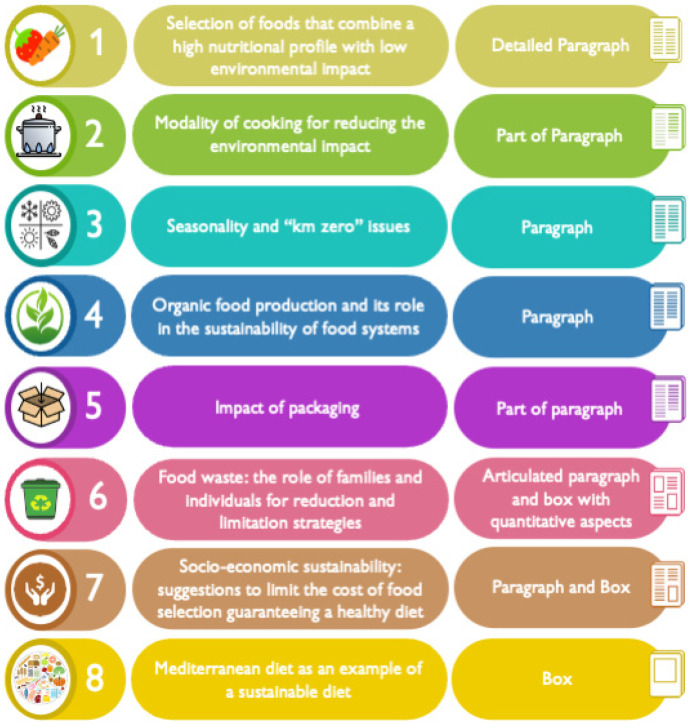
Content of the IDGs’ sustainability chapter (directive 13) and how the subjects were presented.

Figure 1 summarizes the content of the IDGs’ directive 13. Topics were divided up into detailed paragraphs, as part of a section, or addressed more fully in a dedicated box.

**Figure 2:**
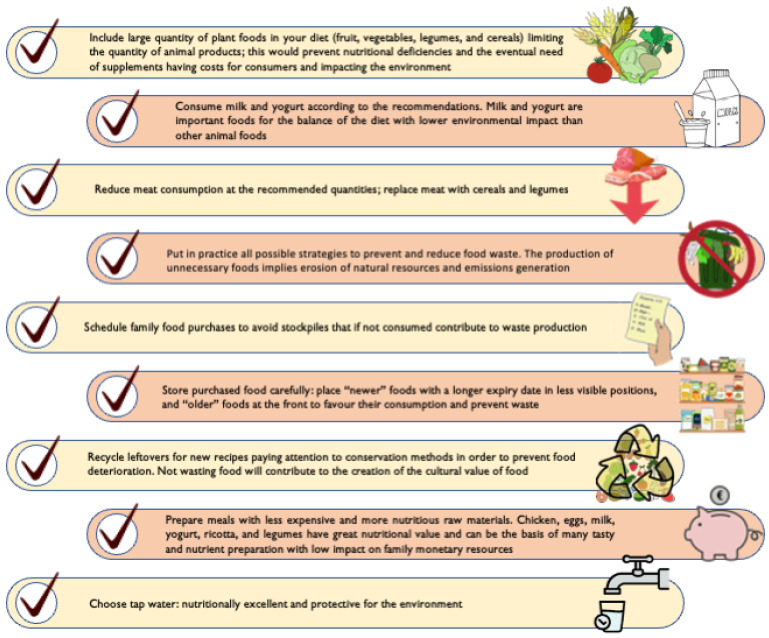
Key recommendations (How to …) from the IDGs’ sustainability chapter–directive 13.

The recommendations of the IDGs’ sustainability chapter are summarised in simple practical “How to …” items at the start of the chapter. The summary is intended to help consumers understand the recommendations without reading the entire chapter. The content of the “How to …” box is summarised in Figure 2.

The IDGs’ sustainability chapter recommendations on food selection discourages items with a two-fold negative impact: on health and the environment. The recommendation is to reduce processed meat and red meat, in favor of poultry, which is less costly and has a smaller environmental impact, and vegetable sources of protein, such as legumes. Regarding fish, the advice is to include a diet of small fish from the Mediterranean Sea, such as anchovies, sardines, mackerel, etc., and reduce consumption of the generally preferred species, such as tuna, salmon, swordfish, etc., that are subject to exploitation and erosion. Consumption of fish from aquaculture is recommended as a strategy to preserve wild resources given the increased quality of farmed fish. As regards fruit and vegetables, the recommendation is to consume seasonal products, limiting the selection of varieties requiring the large use of external input for growing, such as fertilizing, artificial light and heating. It is also recommended to limit the consumption of overseas products.

Food loss and waste were largely addressed, as priority attention was given to these aspects in Italy since the approval of a dedicated law [20] in line with the international commitment to food loss and waste reduction and prevention [21]. Considering the IDGs’ targets, the recommendations were focused on consumers’ behaviors, providing advice on how to plan food purchases, to prepare and store food in light of waste reduction and prevention and as a strategy to save money. It was suggested to avoid buying more than needed, in order to keep food fresh and limit waste. Practical examples were given on how to re-utilize old ingredients or leftovers in new recipes. The availability and economic sustainability of nutritional foods were addressed, providing recommendations for a healthy diet that was also affordable in monetary terms. Examples of ingredients combining a high nutritional value and low cost, such as eggs, poultry, beans, milk and seasonal vegetables, were provided to help the dietary choices of consumers with limited monetary possibilities.

**Figure 3:**
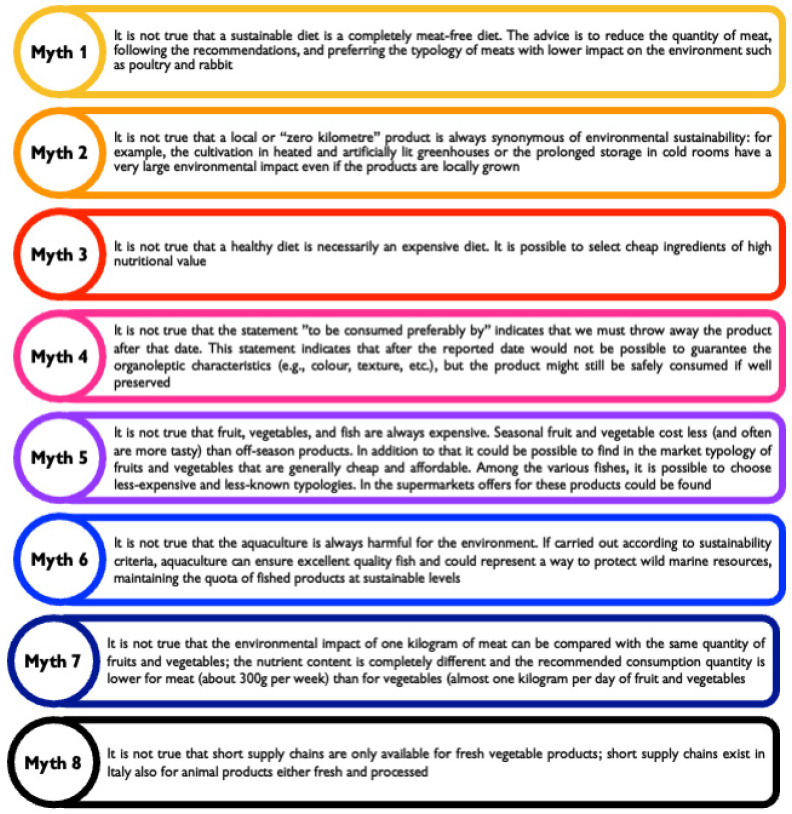
Myths and false beliefs on the sustainable diets.

A list of “myths” and false beliefs was reported at the end of directive 13 to demonstrate that people’s erroneous beliefs could also be addressed in the framework of institutional communication (Figure 3).

In 2019 [3], the Food and Agriculture Organization of the United Nations (FAO) and the World Health Organization (WHO) developed the guiding principles around what constitutes sustainable healthy diets. The guiding principles for sustainable healthy diets are food-based, combine recommendations with environmental, social/cultural and economic sustainability, and need to be translated into clear, non-technical information and messaging to be used by governments and other actors in policy-making and communications. Table 1 shows the level of inclusion and attainment of the 16 guiding principles of a sustainable healthy diet in the IDGs, in the context of sustainability. Although the IDGs were developed before the publication of the 16 guiding principles of a sustainable healthy diet, it is interesting to see that most of the principles were included in the IDGs, either as general recommendations or with a sustainability point of view. Although principles, such as breastfeeding and NCD had the potential to be treated in terms of sustainability, they were only considered in terms of health-promotion. Several guiding principles were considered only in terms of sustainability (e.g., biodiversity, food waste, packaging and cultural aspects of food choices). Three principles were not treated in the IDGs, namely processed foods, antibiotics and hormones for food production and gender issues. The reasons for the non-inclusion of these topics were varied. Food policy in Italy is not specifically addressed to discriminate between processed and non-processed foods, considering the nutritional profile as more relevant than the technological process of production. Further reflection on these aspects should be carried out with a better understanding of the impact and quantitative relevance of processed foods in the overall Italian diet. Antibiotics and hormones were merely hinted at in the safety chapter of the IDGs (directive n. 12), in consideration of the very strict legislation in Italy and Europe on the use of antibiotics and hormones banned since 2005 [22]. Gender aspects, in terms of equality and women’s participation in social life, were not treated in the IDGs, which only touched on aspects of changing nutrient requirements in the various female physiological life stages (e.g., pregnancy, lactation, menopause). This is a gap in the current IDGs that needs to be remedied in further revisions.

### 2.2. Policy Implications on the Inclusion of Sustainability in the IDGs

The treatment of sustainability in the framework of nutritional guidelines is an emerging issue and a relatively new topic since the FBDGs remain predominantly health-focused and often neglect the environmental aspects of diet. However, in the European region, a relevant part (20–30%) of the total environmental impacts of households are associated with food consumption and several initiatives claim that a change in the food systems can address sustainability aspects [23]. As reported by Cambeses-Franco et al., 2022 [24], the FOOD 2030 initiative, in line with the European Green Deal, farm to fork strategy and bio-economy strategy, embraces this transformation with the aim to promote sustainable healthy diets, circularity and resource efficiency, innovation and empowerment of communities. FOOD 2030 highlights that the FBDGs could be drivers for changing and promoting healthy, balanced and sustainable food habits [25].

The IDGs, to some extent, anticipated these general recommendations as their development began in 2013. During the preparation of the IDGs’ sustainability chapter, to avoid assumptions and to limit the bias of personal interpretation, particular attention was given to the selection of recommendations supported by the literature. In the development of recommendations, priority was given to actions possible at the consumer level over those proper to the productive system. The idea was to provide families and individuals with advice for daily choices to encourage behaviors combining the selection of food promoting health and environmental protection.

The IDGs is a consensus document resulting from acceptance by a large scientific and political community. A national task force was established with experts from universities, research bodies, scientific societies and consumer associations. In addition, the document was endorsed by the Ministries of Agriculture, Health, Environment and Education [26]. This revision, for the first time, included the contribution of the Ministry of Environment, which worked specifically on the sustainability chapter. The underlying idea of this involvement was to bring in a point of view to some extent separate to nutritional aspects, with the challenge of combining expertise in different fields. The added value of this multidisciplinary approach is related to the fact that dietary behavior is a strong determinant of food consumption, which represents a key lever for action to transform food systems and vice versa. The involvement of the Italian Ministry of Environment was important to provide recommendations and solutions for transition through a transdisciplinary and multi-level approach addressing the factors influencing dietary behavior and health, together with the environmental and socio-economic impacts of dietary patterns. Ministry of Environment technicians were included in the process of development of the IDGs’ sustainability chapter to approach the development of recommendations with a holistic method considering consumers’ dietary behaviors and food consumption as relevant components of the agri-food-systems able to influence food production. In Europe, the farm to fork strategy [27] and recent studies on an EU food system transition [28,29,30] have embraced this holistic method to avoid sectorial approaches that are limited in terms of objectives or partial sub-systems [31]. The Ministry of Environment’s contribution to directive 13 of the IDGs, concerned the Italian commitment to sustainable development strategies for the implementation of the 2030 Agenda. On the Ministry of Environment’s website, a page dedicated to this joint effort was created to highlight these efforts [32].

The inclusion of sustainability in the IDGs was the driving force behind a series of actions and research projects aimed at increasing the knowledge of the relationship between dietary habits, food consumption and sustainability aspects. The first step was the creation of a dataset merging food consumption data with environmental footprint data, in terms of GHGE emissions. The database of the national food consumption INRAN-SCAI 2005–2006 survey [33,34], linked with the Italian Food Composition Databases [35] and the Food Composition Database for Epidemiological Studies in Italy [36], was combined with the GHGE estimations, providing data on the kg CO_2_ equivalent per each 100g of food items [37]. Through combined database modeling, it was demonstrated that only by aligning consumption with the IDGs’ recommendations would it be possible to achieve a reduction of 50% of GHGEs, with respect to the Italian adult population’s current dietary patterns [37]. The added value of this study was that the reduction of GHGE could be achieved with a dietary pattern that meets dietary requirements for health, without eliminating meat or dairy products, but consuming them according to the recommendations. The IDGs’ dietary advice derived from health and sustainability considerations, and the modeling results showed that the shift in Italian dietary habits, proposed in the IDGs, could result in more environmentally sustainable food consumption patterns. The high level of sustainability of the diet recommended in the IDGs was further demonstrated in the paper by Cambeses-Franco et al., 2022 [38], which compared the carbon footprint (kgCO2eq/person/day) and water footprint (L/person/day) of the planetary diet provided by the EAT-Lancet commission with the Spanish dietary pattern and the recommended dietary patterns resulting from the guidelines of Italy, the Netherlands, the Mediterranean region and America. The results highlighted the best environmental indicators, carbon footprint and water footprint, for the diet recommended in the IDGs. The modeling methodology was also used to demonstrate that among existing school catering menus, it would be possible to select recipes combining a low environmental impact with a high adherence to the IDGs’ recommendations [39,40].

The IDGs’ sustainability chapter largely addressed the topic of food waste, providing a set of recommendations to prevent and reduce food waste at the household level. In Italy, food waste prevention initiatives at the consumer level were linked to food behavior and consumption, and other aspects, such as the promotion of a varied, healthy and sustainable diet, as reported in the IDGs [41]. Since the publication of the IDGs’ sustainability chapter, connections between dietary habits and food waste behavior were further analyzed, showing that consumers who follow the IDGs’ recommendations tend to have a highly preventive attitude towards food waste [42,43].

## 3. Further Steps of Sustainability Inclusion in the Dietary Guidelines

### 3.1. What Methodology Could Be Proposed?

One of the limitations of the Italian approach to the development of sustainability recommendations was that it was carried out without an evaluation of Italian consumers’ awareness of sustainability. A duly designed survey on this topic was carried out in 2022 on a representative sample of Italian consumers with an approved questionnaire to assess Italian consumers’ level of awareness of the environmental impact of food choices, with a specific focus on whether alternative protein to meat, even new generation, would be accepted by Italian consumers [44]. The preliminary results of this work indicate a dichotomous attitude on the part of consumers, who by and large agreed that food consumption can affect the environmental sustainability but wanted to be free from constraints and limitations, in terms of dietary choices. Italian consumers considered other factors (deforestation, use of cars, etc.) as much more relevant to sustainability than food consumption and eating habits [45]. The reduction of meat consumption was strongly recommended in the IDGs’ sustainability chapter. Currently, Italian consumers reported their willingness to replace meat with known foods considered as part of traditional consumption, such as legumes, eggs, fish, cheese and nuts. Moreover, respondents are much less likely to replace meat with novel foods such as, seitan, seaweed, tempeh, jellyfish and other meat substitutes. In addition, Italian consumers reported a strong resistance to the consumption of insects and synthetic meat. As a policy implication, this nationwide country representative assessment could also be used as a benchmark for developing specific recommendations, in consideration of the limits of the inclusion of sustainability in nutritional advice, and could contribute to the maximization of the capacity of the dietary guidelines, to create a healthy food environment [44,46].

Teschner et al., 2021 [47] reported that, besides the general agreement on the need for more sustainable diets, a definition of sustainable dietary recommendations for consumers is still lacking and this is relevant in the development of the dietary guidelines. Consumers’ understanding of sustainability is one aspect of the development of the relative recommendations and their inclusion in the FBDGs. A codified methodology for sustainability was still not available for inclusion in the dietary guidelines, however several experiments are reported that used the optimization methodology. A linear programming analysis is a mathematical approach that can be used to model dietary consumption, based on current dietary patterns with the inclusion of sustainability indicators. The development of the FBDGs with diet optimization has the advantage of controlling the variables and also including sustainability items as elements of the model [48,49], evaluating the complexity of the overall food consumption pattern [50]. Schäfer et al., 2021 [51] reported the results of the optimization methodology to develop the German FBDGs, that included sustainability recommendations, and they considered the flexibility of the mathematical model useful to identify dietary changes in Germany that would take into account diet-health relations, environmental impact and cultural acceptability. Another interesting trial concerning the optimization of dietary patterns to develop the FBDGs that simultaneously meet recommendations for food groups and nutrients and limit, at maximum, the foods with a high environmental impact, was carried out in the Netherlands, combining the optimization of dietary patterns with recommendations, based on expert judgment [52].

In Italy, as previously reported, the optimization of food consumption was used to demonstrate a reduction in the environmental impact of the recommended diet without the exclusion of any food group, including red meat and milk [37]. Until now, we have not used optimization for the IDGs’ development, while the possibility of using the modeling methodology is not definitive or exempt from limitations. Optimization could be a suitable approach to guide the development of recommendations aimed at improving the sustainability of diets considering all of the characteristics of diets, to find solutions for nutritional, environmental, and economic aspects mitigating any possible incompatibilities among dimensions [53]. However, an important aspect that needs to be taken into account in the FBDGs’ development, is the cultural acceptability of foods as part of the sustainability of the diet. The selection of sociocultural indicators is subjective and depends on the availability of data, which represents a limit for the application of the optimization approach. As demonstrated in the abovementioned work on consumers’ acceptability in Italy of meat substitutes, a recommendation that includes an “exotic” meat alternative would be unlikely to be accepted, even if it were the best solution in terms of environmental impact [45]. As reported by Perignon and Darmon (2022), the identification of indicators for assessing the cultural acceptability and the availability of reliable data on these aspects are difficult and challenging [53].

The general recommendations for developing DGs that could be proposed on the basis of the first experience carried out in Italy would be a combined approach of the optimization of food consumption, coupled with consumer acceptance studies, at least for particularly innovative topics. In addition to these quantitative and measurable approaches, we still consider expert judgments important as the final contribution in developing the recommendation, as reported in the case of the Netherlands that combined advice from scientists and dietitians with consumer input [52]. Expert judgment is still the most critical point of the method, as long as the background, competencies and in some cases convictions of the scientists involved in the process, are counterbalanced by a large expert panel from the different areas of expertise.

### 3.2. What Is the Sustainability Level in Italy?

The main limitation of the IDGs is related to the existing gap between dietary recommendations and actual consumer behavior, indicating a generally poor compliance with the IDGs [18]. In Italy, a detailed plan for developing strategies to assist behavior change is still lacking and this is particularly true for recommendations on a sustainable dietary pattern that have only recently been developed [18]. On this topic, it is interesting to note the outcome of the evaluation of the food sustainability index (FSI) measuring the sustainability of food systems in countries around three key pillars: food loss and waste, sustainable agriculture and nutritional challenges. One of the aspects with the highest score in the nutritional pillar is to have dietary guidelines that have been updated in the past 5 years and explicitly mention sustainability or the environment [54]. In the 2021 data, Italy ranked 16th out of the 78 countries included in the evaluation, with a very high level for food loss and waste ranking at 2/78. However, in terms of sustainable agriculture (ranked 53/78) and nutritional challenges (32/78), the situation is far from optimal. The nutritional challenges of this evaluation deserve comment in consideration of the fact that besides Italy’s high scores relating to a healthy life expectancy, low nutrient deficiencies and affordability of a healthy and sustainable diet, critical aspects are reported for physical activity, the prevalence of over-nourishment, the environmental impact of dietary patterns, diet composition and policy nutritional response. It should be pointed out that the top performers in the nutritional challenges’ pillar include the EU countries of Sweden, Denmark, France, the Netherlands, Luxembourg and the United Kingdom, which are already developing policies to strengthen the link between diet and sustainability. The FSI has been evaluated periodically since 2016. Keeping trace of this index could be a way to monitor the impact of ongoing campaigns promoting sustainability in food choices, including the IDGs’ recommendations.

### 3.3. The Role of the IDGs’ Sustainability Chapter in the SDGs’ Achievement

The 2030 Agenda for Sustainable Development, adopted by all United Nations member states in 2015, is a common cooperation framework to promote sustainable development. The Agenda is articulated in 17 Sustainable Development Goals (SDGs) that include actions and measurable targets to improve health and education, reduce inequality, promote economic growth, address climate change and the preservation of oceans and forests [5]. The SDGs have a transversal nature and are applicable in developed and developing countries. SDG 2 (‘end hunger, achieve food security and improved nutrition and promote sustainable agriculture’) is the only SDG that mentions ‘nutrition’, which is, however, directly or indirectly an important cross-cutting issue in all SDGs, [19]. The IDGs can contribute to achieving the Sustainable Development Goals (SDGs) by the promotion of a dietary pattern protective of human health and the environment. According to Grosso et al., (2020) [19], there are multiple direct and indirect pathways by which policies to promote a healthy diet for people and the environment support the 17 SDGs. The potential contribution of the IDGs’ directive 13 to the achievement of SDGs (Table 2) is an interesting exercise to evaluate how far Italian recommendations on sustainability reach the pre-fixed objectives of the 2030 Agenda identifying the areas of improvement that could be addressed in the implementation and dissemination activities of the IDGs. The strengths and weaknesses of the IDGs’ sustainability recommendations supporting the SDGs were reported.

## 4. Discussion

People’s awareness of the environmental effects of food production and consumption is still limited, despite increasing evidence of the huge impact on the ecosystem of consumers’ dietary habits [67]. The connections between nutrition and environmental aspects in the FBDGs are important to achieving sustainability objectives. In addition to that, allowing for differences from country to country, a high adherence to the recommended diets was associated with better nutritional and environmental indicators [24]. Lifestyle and behavior changes are important objectives of nutrition education. They can also help reduce the environmental impact of the food system, reduce food loss and waste, and boost sustainable resource use. Several countries have started to incorporate sustainability aspects into nutrition education programs addressed to consumers. Dietary guidelines have policy and programmatic implications, hence the integration of recommendations that promote specific food practices and choices would maximize the advice on sustainability. Such recommendations include for example: having a mostly plant-based diet, focusing on seasonal and local foods, reduction of food waste, consumption of fish from sustainable stocks, and reduction of red and processed meat, highly-processed foods and sugar-sweetened beverages [26]. At the European Level, the guidance on sustainability was mainly provided in terms of recommendations for the selection of local seasonal products and reducing waste; however, frequently this advice did not make explicit mention of the environmental aspects [68]. The IDGs provided practical suggestions aimed to improve consumer behaviors, in terms of environmental protection through health-promoting recommendations showing that it is possible to pass on practical advice to consumers, aimed to improve their behavior in terms of sustainable development [18].

There are still limitations to this approach. In the IDGs, the sustainability recommendations were developed without an evaluation of Italian consumers’ considerations of sustainability [44]. As reported by Rossi et al., 2022 [18], the sustainability of diet is an aspect that is still not completely exploited, in which the risk of bias and “personal” interpretation is also possible, given the limited sources of information, based on consensus documents. In the IDGs, the strategy of prioritizing recommendations for health protection and combining them with environmental aspects avoided confusion, appearing sufficiently coherent for such a public health nutrition document [18]. Limitations of this approach were largely discussed at coordination meetings in the process of the IDGs’ development. During the IDGs’ preparation, sources of information were limited by the low availability of consensus documents, and an overall estimation of the different aspects of sustainability of the food system is still lacking [69]. Another limitation to the development of the sustainability of diet in the IDGs, was related to the fact that the commission included nutrition experts with an under-representation of environmental sustainability competencies. In a modern approach, nutritional recommendations should consider the environmental implications of food choice, and the development of nutritional recommendations are tasks for community nutrition specialists and public health experts [44]. A multidisciplinary approach to the development of nutritional recommendations is important for the wide range of aspects belonging to different disciplines that should be considered together in order to identify possible solutions for the transition towards a healthy and sustainable diet.

## 5. Conclusions

The integration of recommendations that promote specific food practices and choices while also addressing the ecological dimensions of nutrition, would represent an efficient strategy to contribute to the reshaping of the current food systems towards the provision of health-promoting products, that are less impacting to the environment. In the IDGs, it was possible to show that there is also the chance of transmitting to consumers practical recommendations aimed to improve their behavior, in terms of sustainable development.

The interrelationships between nutrition and the environment in the FBDGs are central to achieving sustainability objectives. The IDGs can contribute to attaining the SDGs considering their potential multiplicative health and social impacts, and developing policy actions to promote a dietary pattern that protects people’s health and safeguards the planet.

The present general structure of directive 13 of the IDGs and its methodology of development should be considered a starting point for future revisions. DGs in Italy should be structured with a combined approach, coupling the optimization of food consumption with consumer acceptance studies, at least for particularly innovative topics, such as sustainability aspects. However, besides these quantitative and measurable approaches, expert judgments remain important and advisable as the final contribution in developing recommendations.

The sustainability of diet is an aspect of the environmental impact of food production that is not yet completely mastered, and the risk of bias and “personal” interpretation is still high. At the time of the IDGs’ preparation, sources of information were limited with few consensus documents available. Over time, more references can be found, however an overall estimation of the different aspects of sustainability of the food system is still lacking. All of these elements need to be taken into account considering that the incorporation of sustainability issues into food policies and consumer education programs is advisable and increasingly common in many countries.

## Figures and Tables

**Table 1:** The 16 guiding principles of a sustainable healthy diet in the IDGs regarding sustainability. Key to symbols: 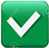 included; 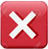 not included.

Related to:	Principle Number and Short Name [13]	Sustainable Healthy Diets: Guiding Principles [3]	In the IDGs	In the IDGs Regarding Sustainability
HEALTH ASPECTS	1. Breastfeeding	An start early in life with the early initiation of breastfeeding, exclusive breastfeeding until six months of age, and continued breastfeeding until two years and beyond, combined with the appropriate complementary feeding.	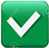	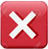
	2. Food processing	Based on a great variety of unprocessed or minimally processed foods, balanced across food groups while restricting highly processed food and drink products.	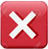	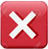
	3. Plant-based foods	Include wholegrains, legumes, nuts and an abundance and variety of fruits and vegetables.	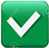	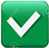
	4. Animal-based foods	Can include moderate amounts of eggs, dairy, poultry and fish; and small amounts of red meat.	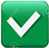	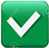
	5. Drinking water	Include safe and clean drinking water as the fluid of choice.	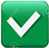	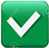
	6. Nutritionally adequate	Adequate (i.e., meeting but not exceeding needs) in energy and nutrients for growth and development, and meet the needs for an active and healthy life across the lifecycle.	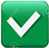	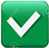
	7. NCD risk	Consistent with the WHO guidelines to reduce the risk of diet-related NCDs, and ensure health and wellbeing for the general population.	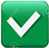	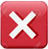
ENVIRONMENTAL ASPECTS	8. Foodborne disease	Contain minimal levels or none, of pathogens, toxins and other agents that can cause foodborne diseases.	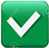	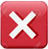
	9. Environmental impacts	Maintain greenhouse gas emissions, water and land use, nitrogen, phosphorus application and chemical pollution within set targets.	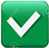	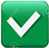
	10. Biodiversity	Preserve biodiversity, including that of crops, livestock, forest-derived foods and aquatic genetic resources, and avoid overfishing and overhunting.	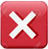	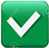
	11. Antibiotics and hormones	Minimize the use of antibiotics and hormones in food production.	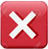	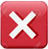
	12. Food packaging	Minimize the use of plastics and derivatives in food packaging.	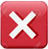	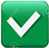
	13. Food waste	Reduce food loss and waste.	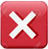	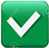
SOCIO-CULTURAL ASPECTS	14. Culture	Build on and respect local culture, culinary practices, knowledge and consumption patterns, and values on the way food is sourced, produced and consumed.	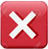	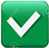
	15. Accessibility	Accessible and desirable.	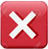	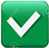
	16. Gender impact	Avoid adverse gender-related impacts, especially with regard to time allocation (e.g., for buying and preparing food, water and fuel acquisition)	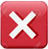	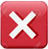

**Table 2:** Policies and recommendations of the IDGs’ sustainability chapter contributing to SDGs’ achievements.

Sustainable Developing Goal	Connection with Nutrition	Recommendations of the Sustainability Chapter of IDGs’ Supporting the SDG
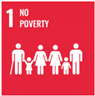	Poverty limits access to sufficient food intake with consequent difficulties in covering nutrient recommendations [19].	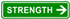 In the sustainability chapter of guidelines, it was shown that a healthy diet could also be low-cost. Recommendations were provided for cheap nutritious foods, such as eggs, poultry, beans, milk and seasonal vegetables, to help consumers with limited monetary resources. 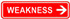 These aspects were poorly addressed globally in the IDGs.
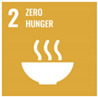	Unsustainable food production causes all forms of malnutrition, including overweight and obesity that represent an outcome of malnutrition, in terms of an unbalanced and excessive food intake [55].	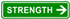 Prevention of all forms of malnutrition is one of the main objectives of the IDGs and the sustainability recommendations were provided to reorient agriculture and food supply policies to be in line with dietary recommendations. 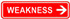 These aspects were addressed in more depth in other directives than in directive 13 of the IDGs.
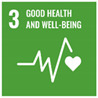	Healthy and sustainable nutrition may reduce premature death and the occurrence of non-communicable diseases [56].	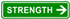 The recommendations of directive 13 of the IDGs combined health promotion with environmental safeguards. As an example, the promotion of fruit and vegetables was expressed as “increase the consumption of fruit and vegetables selecting local and seasonal products with a low use of external input for production” (human health and environmental protection). 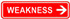 These aspects were addressed in more depth in other directives than in directive 13 of IDGs.
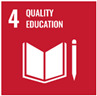	Socio-economic aspects including education are strong determinants of food choices, the nutritional quality of the diet and people’s nutritional status [57].	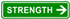 School programs, teacher training and educational laboratories used the IDGs as a tool for developing learning materials. All of these educational products included sustainability aspects in addition to dietary recommendations. 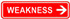 Quality of education was not the core topic of the IDGs.
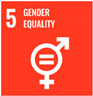	According to the global gender gap index, in 2018, Italy ranked 70th among 149 countries, in terms of women’s participation in economic and political activities, and access to education and health [58]. In addition to that, physical activity participation in Italy reflects a gender bias with males more likely to be active than females [59].	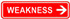 The IDGs’ sustainability chapter, as structured in the present edition of the guidelines, did not approach the gender aspect. A reflection on how to include gender issues in the future development of sustainability recommendations needs to be carried out. A possible area is the promotion of physical activity that would contribute to the sustainability of mobility policies, privileging safe and affordable transport systems (walking, cycling, etc.), especially in women, a population group in which a sedentary attitude is common.
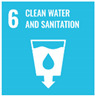	Among European countries, Italy has the highest number of natural mineral water brands [60]. This bottled-water market heavily impacts the environment, considering that more than 3 L of water was needed to provide consumers with 1.50 L of drinking water from six Italian brands, compared with Italian tap water [61].	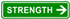 Access to safe drinking water is not an issue in Italy. The importance of tap water consumption in consideration of its nutritional value and safety, was largely addressed in directive 13 of the IDGs. The false belief that tap water is unhealthy was also reported in the water-dedicated chapter of the IDGs.
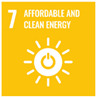	The transition towards a mostly sustainable food production will also depend on a reduction of greenhouse gas emissions, ensuring food safety [7].	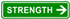 In the IDGs’ sustainability chapter, these aspects were put forward in recommendations of the selection of fruit and vegetables in line with seasonality, limiting the products that require high fertilizing, artificial light and heating, or overseas products. in the IDGs, foods with low greenhouse gas emissions were recommended for equal nutritional value. 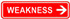 Production system and energy aspects were addressed, only to a limited extent in the IDGs,
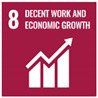	Sustainable primary production and nutrition safety could be pursued with a proper economic transformation [19].	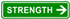 In directive 13, specific recommendations for low socioeconomic groups were provided, to target the most in need. The importance of so-called nutrition-sensitive production that promotes nutritionally rich foods contributing to dietary diversity for all population groups was highlighted. 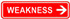 Economic productive subjects were not predominantly treated in the IDGs.
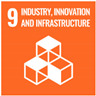	Inclusive and sustainable industrialization, together with innovation and infrastructure, are essential for food production and food safety. Thus, not only the quantity of food produced is relevant, but also how food is produced, harvested, processed, distributed, marketed, disposed of and eaten [55].	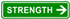 The IDGs’ sustainability chapter encouraged a healthy dietary pattern with a low reliance on animal-source foods, characterized by the reduction of food waste. 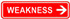 The production sectors (agriculture, industry, distribution) were not sufficiently involved in this process. A further improvement of the IDGs, especially in terms of sustainability, should take these aspects into account as well.
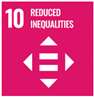	Social, economic and educational inequalities strongly influence people’s diets. The poor are affected the most and suffer the greatest repercussions from dietary inequalities due to a lack of access, availability and affordability of nutritious foods [56].	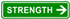 Two aspects of directive 13 of the IDGs could be seen in light of the reduction of inequalities, the focus on the cost of diet and food waste issues. Recommendations were provided for a nutritious and affordable diet, in terms of cost. Food waste reduction and prevention were also addressed as strategies for saving money. 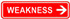 Inequalities were poorly addressed in the present revision of the IDGs.
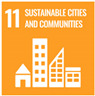	According to Barbour et al., 2021 [62], urban local government authorities have a key role in implementing policies that could optimize the food supply chain, to assist the local population with healthy and sustainable diet-related practices.	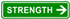 In the IDGs’ sustainability chapter, an upgrade of the guidelines to enhance knowledge about the environmental implications of dietary choices was proposed, especially in residential communities. 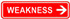 Several aspects are still missing and the role of city and local authorities in promoting the health and environmental protection of the recommended diet-related practices is something that needs further development.
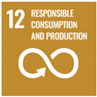	Individual food choices have impacts that resonate far beyond themselves: diet reflects larger systemic issues that affect population health, sustainability and justice [63,64].	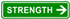 Particularly relevant for the achievement of this goal was the space given to food waste prevention and reduction at the household level, providing practical and simple recommendations for action (e.g., management of food purchase and storage, and reuse of leftovers). Label issues were stressed, in particular the correct utilization of “best before” advice.
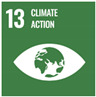	Food systems impact the environment and contribute to climate change. The increasing demand for animal-sourced foods represents a threat to the environment, contributing to biodiversity loss and deforestation, and representing a concern for animal welfare [55].	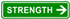 The main recommendation of the IDGs’ sustainability chapter was the reduction of consumption of red and processed meat, maximizing the quota of plant foods, and the intake of proteins from vegetable sources (e.g., legumes). This recommendation combines human health promotion and environmental protection. 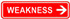 Biodiversity loss, deforestation, and animal welfare were not directly addressed in the IDGs.
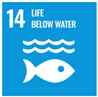	Aquaculture reduces hunger and improves nutrition; overfishing largely impacts on environment, limiting biodiversity and eroding natural resources [19]	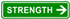 In directive 13 of the IDGs, it was recommended not to demonize the consumption of fish from aquaculture as a strategy to preserve wild resources, in consideration of the increased quality of farmed fish (health protection) and considering that aquaculture production with sustainability criteria does not harm the environment (sustainability consideration).
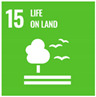	The reduction of animal protein consumption and increase in vegetable protein intake is largely demonstrated to be protective for humans and the environment [7].	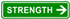 This aspect was largely addressed in the IDGs’ sustainability chapter, affirming the importance of a mostly plant-food diet alongside the intake of animal foods, following the principles of the Mediterranean Diet.
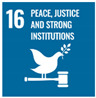	Social protection and strong institutional support are important means to protect people from food insecurity, as demonstrated in the recent periods of economic recession in Europe. Food insecurity could become persistent in countries with low social protection levels [65].	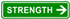 In the IDGs, the population groups most at risk for food insecurity were identified: women, large families with limited income, very poor people and individuals with low education. These groups need to be subject to specific evidence-based policies and measures for protection. 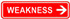 In addition to the effort in the identification of recommendations tailor made for these population groups, the capacity of the IDGs to reach them needs to be demonstrated and more efforts should be made to contribute to these aspects.
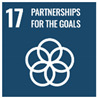	Single dietary intervention could not address the complexities of the current food system. There is a need for complementary and synergistic approaches. Basic and applied nutritional research and innovation should result from strongpartnerships between sectors able to develop evidence-based priorities for dietary policies [66]	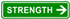 The IDGs’ sustainability chapter described a way to strengthen the usual partnership between the world of production and the health sector. The inclusion of environmental aspects in dietary recommendations would create a cultural bridge between so far unrelated worlds. 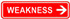 Efforts should be made to increase synergies in actions that could improve the impact of policies.

## Data Availability

The archived data and all elaboration and analysis generated and used for the presentation of results in this study are fully available on request from the author concerned.
